# Magnoflorine Suppresses MAPK and NF-κB Signaling to Prevent Inflammatory Osteolysis Induced by Titanium Particles *In Vivo* and Osteoclastogenesis *via* RANKL *In Vitro*

**DOI:** 10.3389/fphar.2020.00389

**Published:** 2020-04-02

**Authors:** Zhenyu Sun, Junkai Zeng, Wenjuan Wang, Xinlin Jia, Qiang Wu, Degang Yu, Yuanqing Mao

**Affiliations:** Shanghai Key Laboratory of Orthopaedic Implants, Department of Orthopaedic Surgery, Shanghai Ninth People’s Hospital, Shanghai Jiao Tong University School of Medicine, Shanghai, China

**Keywords:** magnoflorine, aseptic loosening, inflammation, osteoclast, mitogen-activated protein kinase signaling, NF-κB signaling

## Abstract

Wear particles that detach from the surface of prostheses induce excessive activation of osteoclast and immoderate release of inflammatory cytokines that lead to peri-implant osteolysis and aseptic loosening. In this work, we investigated whether magnoflorine, a quaternary aporphine alkaloid extracted from the Chinese herb Magnolia or Aristolochia, could effectively inhibit inflammatory calvarial osteolysis caused by titanium particles in mouse models, inflammatory response as well as osteoclastogenesis *in vitro* mediated *via* receptor activator of NF-κB ligand (RANKL). Micro-computed tomography and histological examination of mice treated with magnoflorine revealed fewer resorption pits, less osteoclasts formation and inflammatory cytokine expression. Moreover, *in vitro* differentiation of osteoclasts and bone resorption as well as titanium particle-induced inflammatory response were dose-dependently inhibited by magnoflorine. These were accompanied by reduced transcription of osteoclast-specific genes encoding tartrate-resistant acid phosphatase (TRAP), V-ATPase d2, c-Fos, cathepsin K, nuclear factor of activated T cells (NFAT) c1, and calcitonin receptor (CTR). Further research on mechanism showed that the inhibition of phosphorylation of TAK1 and subsequent activation of MAPK and NF-**κ**B signaling pathways were found to mediate the suppressive effects of magnoflorine. Collectively, these results suggested that magnoflorine treatment could effectively prevent peri-implant osteolysis due to wear debris as well as other diseases caused by chronic inflammation and excessive osteoclast activation.

## Introduction

Severe hip joint diseases, such as osteoporotic fracture, aseptic necrosis of the femoral head, osteoarthritis, end-stage rheumatic arthritis, and other arthritic diseases, often require total hip arthroplasty to alleviate pain, increase the range of motion, and improve the patient’s quality of life (Van Citters et al., 2014). Even though great efforts have been expended in ameliorating the efficacy of THA, peri-prosthetic osteolysis (PPO) and subsequent aseptic loosening after long-term use of artificial joints remain to be the principle cause of arthroplasty failure (Abu-Amer et al., 2007; Patel et al., 2015). To date, considerable evidence indicates that PPO initiates and develops after wear debris accumulates at the bone–implant interface due to biological or mechanical responses (Gallo et al., 2014; Stratton-Powell et al., 2016). Generally, immune cells based on macrophages are recruited to this area, and afterwards the phagocytosis of particles by macrophages stimulates the release of various inflammatory cytokines (Dumbleton et al., 2002; Tian et al., 2014). As a result, osteoblasts and bone marrow stromal cells are directly and indirectly induced to express receptor activator of nuclear factor-κB (NF-κB) ligand (RANKL) (Holding et al., 2006), which binds to and activates its cognate receptor RANK on osteoclast precursors to enhance bone resorption and osteoclast formation *via* activating plenty of downstream signaling pathways, of which the NF-κB and mitogen-activated protein kinase (MAPK) signaling pathways are of great significance (Feng and Teitelbaum, 2013; Yan et al., 2017). Thus, inflammatory microenvironment and overactivation of osteoclastogenesis play vital roles in peri-prosthetic bone loss and subsequent prosthetic loosening and instability.

There are two main approaches for improving the clinical outcomes of total joint prostheses: i) to fabricate implanted prostheses from more biocompatible materials and/or modify their surfaces to minimize wear particles and provide better integration into host bone, and ii) to identity and administer compounds that suppress the macrophage-based inflammatory response induced by wear particles, osteoclast differentiation, and bone resorption. To this end, we identified a natural compound called magnoflorine isolated from the Chinese herb Magnolia, or Aristolochia (Li and Wang, 2014). Magnoflorine is commonly used as an anxiolytic chemical but also exhibits anti-inflammatory (Guo et al., 2018), antifungal (Chen et al., 2009; Kim et al., 2018), antidiabetic (Patel and Mishra, 2012), antioxidant (Zheng and Wang, 2001), anticancer (Min et al., 2006), and antihypertensive (El Tahir, 2008) properties. For example, it was reported to be relevant to inflammation as showed to dose-dependently reduce the secretion of proinflammatory cytokines and protect against lipopolysaccharide-induced inflammation in acute lung injury (Guo et al., 2018). This compound also acts as an α-glucosidase inhibitor *in vitro* and *in vivo* and exerts excellent anti-hyperglycemic effects in rats (Patel and Mishra, 2012). Moreover, magnoflorine has been showed to play a role in lowering arterial blood pressure and induced hypothermia in laboratory animals (El Tahir, 2008). However, to our knowledge, whether magnoflorine can adjust bone metabolism by inhibiting osteoclast differentiation and inflammatory response dominated by macrophages, as well as attenuate inflammatory osteolysis mediated by titanium particle have not yet been investigated. Besides, the potential mechanisms of magnoflorine in RANKL-mediated osteoclastogenesis still remain unclear.

Thus, we performed the experiments presented in this study to i) clarify the potential therapeutic benefits of magnoflorine on Ti particle-induced peri-prosthetic osteolysis, ii) investigate whether magnoflorine inhibits the macrophage-dominated inflammatory response and RANKL-induced osteoclastogenesis, and iii) reveal the mechanisms underlying the influences of magnoflorine on osteoclast formation and function. The results presented here suggest a promising strategy to prevent PPO and prosthesis loosening thereby extending the life span of joint prostheses.

## Materials and Methods

### Chemicals, Reagents, and Antibodies

Magnoflorine [C_20_H_24_NO_4_; MW, 342.41; purity, ≥98% (Meilun, Dalian, China)] was dissolved in dimethyl sulfoxide. Recombinant murine M-CSF and RANKL were from R&D Systems (Minneapolis, MN, USA). Fetal bovine serum and alpha-modified minimal essential medium (α-MEM) were supplied by Gibco-BRL (Sydney, Australia). The staining kit for tartrate-resistant acid phosphatase (TRAP) was obtained from Sigma-Aldrich (St. Louis, MO). The assay for cell viability, cell counting kit-8 (CCK-8), was from Dojindo Molecular Technology (Japan). Primary antibodies targeting extracellular signal-regulated kinase (ERK) and its phosphorylated form (phospho-ERK; Thr202/Tyr204), p38 and phospho-p38 (Thr180/Tyr182), TAK1 and phospho-TAK1, c-Jun N-terminal kinase (JNK) and phospho-JNK (Thr183/Tyr185), NF-κB (p65) and phospho-NF-κB, NF-κB inhibitor alpha (IκBα) and phospho-IκBα, c-Fos, β-actin and histone H3 (as internal controls), and appropriate secondaries conjugated to fluorescent dyes were from Cell Signaling Technology (Cambridge, MA). The primary antibody recognizing nuclear factor of activated T cells, cytoplasmic 1 (NFATc1) was supplied by Absin Bioscience Inc. (Shanghai, China). The Prime Script RT reagent kit and SYBR^®^ Premix Ex Taq™ II were from Takara Biotechnology (Otsu, Japan).

### Preparation of Ti particles

Commercial pure Ti particles (Alfa Aesar, Haverhill, MA, USA) were <20 µm in diameter. They were baked for 8 h at 180°C and then washed in 75% ethanol for 48 h for endotoxin removal before resuspension in sterile phosphate-buffered saline (PBS) at 0.3 g/ml and storage at 4°C. Ti particles were confirmed to be free of endotoxins *via* a Limulus amebocyte lysate assay.

### Ti Particle-Induced Murine Model of Calvarial Osteolysis

The mouse model of calvarial osteolysis used in these studies was described previously (Tian et al., 2014; Wu et al., 2015). All experimental procedures were performed in strict accordance with the guidelines for Care and Use of Laboratory Animals and were approved by the Animal Care Committee of Shanghai Jiao Tong University. Twenty-four 6–8-week-old male C57BL/6 mice (Shanghai Slac Laboratory Animal Company) were housed under specific-pathogen-free conditions and placed into four groups of six mice each: those that underwent a sham operation and were treated only with PBS (sham group), those treated with Ti particles in PBS (vehicle group), those treated with Ti particles and 2 mg/kg magnoflorine (low‐dose magnoflorine group), and those treated with Ti particles and 5 mg/kg magnoflorine (high‐dose magnoflorine group). During the surgeries, the middle sutures of the calvarias received coatings of 30 mg sterilized Ti particles, which were applied under the periosteum. Beginning 2 days later, PBS or magnoflorine was injected under the scalp every other day for 14 days. Experimental procedures did not result in any adverse effects or mortality. After the last treatment, the mice were killed by an overdose of anesthetic, and the calvarias were harvested and fixed in 4% paraformaldehyde.

### Micro-CT Scanning

The degree of calvarial osteolysis was analyzed *via* high-resolution micro-CT (μCT-100; SCANCO, Brüttisellen, Switzerland) with a 10-µm isometric resolution, 300-ms exposure time, and 70 kV, 200 μA X-ray energy. Regions of interest (ROI) in reconstructed images were defined as a square around where the coronal and sagittal midline suture intersect. Bone mineral density, the bone-to-tissue volume ratio (BV/TV, %), the number of pores, and the percentage of total porosity of each sample were measured in the ROI as previously reported (Wedemeyer et al., 2007).

### Histological Staining and Histomorphometric Analysis

Paraformaldehyde-fixed calvarias were decalcified in 10% (w/v) EDTA (pH 7.4) for approximately 2 weeks before they were embedded in paraffin. Hematoxylin and eosin (H&E) and TRAP staining and immunohistochemistry for tumor necrosis factor alpha (TNF-α) and interleukin 1 beta (IL-1β) were performed and imaged by microscopy (Leica Microsystems, Wetzlar, Germany). The number of multinucleated TRAP-positive osteoclasts, the percentage of osteoclasts surface per bone surface (OcS/BS %) as well as amount of TNF-α- and IL-1β- positive cells were quantified with ImageJ software.

### Bone Marrow-Derived Macrophage (BMM) Isolation and Culture

Primary BMMs were isolated from the femurs and tibiae of male C57/BL6 mice (6 weeks of age) as previously described (Tian et al., 2014; Zhai et al., 2014; Wu et al., 2015) and cultured in complete α‐MEM (containing 10% fetal bovine serum and 100 U/ml penicillin/streptomycin) supplemented with 30 ng/ml M‐CSF. The cells were maintained in a humidified environment of 5% CO_2_ and 37°C for another 5–6 days to obtain BMMs.

### Cell Viability/Cytotoxicity Assay

For CCK-8 assays, 8 × 10^3^ BMMs cells were seeded in triplicate in a 96-well plate and cultured for 48, 72, or 96 h with complete α-MEM supplemented with M-CSF (30 ng/ml) and increasing concentrations of magnoflorine (0, 12.5, 25, 50, 100, 200, 400, and 800 μM). Then, 100 μl fresh medium containing 10 μl CCK-8 solution was added to each well. The plates were incubated at 37°C for another 2 h before the optical density at 450 nm wavelength was measured (with 630 nm serving as the reference wavelength) with a microplate reader. Cell viability is expressed as a percentage relative to vehicle-treated control cells (100%).

### Osteoclast Differentiation and TRAP Staining Assay

To assess osteoclast differentiation, 1.0×10^4^ BMMs were seeded in triplicates in 96-well plates and cultured in complete α-MEM supplemented with M-CSF (30 ng/ml), RANKL (50 ng/ml), and magnoflorine (0, 50, 100, and 200 μM). The media was changed every other day until multinucleated pancake-shaped osteoclasts were observed (day 5–7). After fixing for 20 min with 4% paraformaldehyde, the cells were incubated in TRAP staining solution for 1 h at 37°C. Multinucleated cells which were positive for TRAP staining and contained more than three nuclei were identified as osteoclasts. Digital images were captured with an optical light microscope (Olympus, Tokyo, Japan), and the numbers of osteoclasts and the areas of cell spread were then quantified using Image J software (NIH, Bethesda, MD, USA).

### Podosome Actin Belt Immunofluorescence

To observe immunofluorescence staining of podosome actin belt, BMMs were treated consistently with the TRAP staining assay as previously described. Briefly, after mature osteoclasts were formed, the cells were fixed for 20 min with 4% paraformaldehyde, and then permeabilized for 5 min with 0.2% Triton X in PBS. Cells were then incubated for 30 min in darkness with rhodamine-conjugated phalloidin diluted in PBS containing 1% bovine serum albumin (BSA). After extensively rinsing with PBS, cells were counterstained for 5 min with 4′, 6-diamidino-2-phenylindole (DAPI) to visualize cell nuclei. A fluorescence microscope (Leica Images) was used to acquire images, and the numbers and spread areas of podosomal actin belts were quantified with Image J software.

### Resorption Pit Formation Assay

BMMs (1.5 × 10^4^ cells/well) were plated in triplicate in Osteo assay surface plates (Corning, NY) coated with calcium phosphate to mimic bone. The cells were incubated in complete α-MEM containing M-CSF (30 ng/ml), RANKL (50 ng/ml), and magnoflorine (0, 50, 100, and 200 μM). On day 7 when mature osteoclasts were observed, the plates were rinsed with a 5% sodium hypochlorite solution to remove adherent cells from the bottle of plates, and air dried. The resorption pits were imaged with a BioTek Cytation 3 Cell Imaging Reader (BioTek, Winooski, VT), and the resorption pit area was quantified with Image J software; the values are presented as ratios relative to the control (0 µM magnoflorine).

### Particle-Mediated Inflammatory Response *In Vitro*

To assess Ti particle-mediated inflammatory response *in vitro*, quantitative real-time PCR and enzyme-linked immunosorbent assay (ELISA) were performed to detect the mRNA and protein levels of pro-inflammatory cytokines, respectively. 3 × 10^5^ cells/well BMMs were seeded into a 6-well plate and cultured in complete α-MEM supplemented with 30 ng/ml M-CSF. After adhering overnight, cells were stimulated for 24 h with 0.1 mg/ml Ti particles and magnoflorine (0, 50, 100, and 200 μM). Then, the supernatants were harvested for ELISA analysis, and total RNA was isolated from the adherent cells for real-time PCR analysis.

### ELISA

After culturing for 1 day, culture medium from Ti particle-exposed cells was harvested, centrifuged for 10 min at 2,500 rpm and then stored at -80°C until used for subsequent analyses. Murine ELISA kits were used to measure the concentrations of TNF-α, IL-1β, as well as IL-6 per manufacturer’s instructions. Absorbances at 450 nm were recorded with a microplate reader.

### RNA Extraction and Quantitative Real-Time PCR Analysis

For osteoclast differentiation experiments, total RNA was extracted from BMM-derived osteoclasts cultivated for 5 days with 30 ng/ml M-CSF, 50 ng/ml RANKL in the absence or presence of diﬀerent doses of magnoflorine (50, 100, and 200 μM) by using Axygen RNA Miniprep Kit (Axygen, Union City, CA, USA) per manufacturer’s directions. For inflammatory response evaluation, the total RNA was extracted as mentioned above. One milligram of total RNA was reversed transcribed with a PrimeScript RT reagent kit (TaKaRa Biotechnology), and the complementary DNAs (cDNAs) served as the template for qRT-PCR assay with the TB Green Premix Ex *Taq* kit (TaKaRa Biotechnology). All reactions were performed in triplicates and were normalized to the housekeeping gene *Gapdh*. The primer sequences (forward and reverse, 5′ to 3′) based on mouse sequences were as follows: GAPDH, GGTGAAGGTCGGTGTGAACG and CTCGCTCCTGGAAGATGGTG; NFATc1, TGCTCCTCCTCCTGCTGCTC and GCAGAAGGTGGAGGTGCAGC; cathepsin K (CTSK), GGGAGAAAAACCTGAAGC and ATTCTGGGGACTCAGAGC; V-ATPase d2, AAGCCTTTGTTTGACGCTGT and TTCGATGCCTCTGTGAGATG; TRAP, CTGGAGTGCACGATGCCAGCGACA and TCCGTGCTCGGCGATGGACCAGA; DC-STAMP, AAAACCCTTGGGCTGTTCTT and AATCATGGACGACTCCTTGG; c-Fos, CCAGTCAAGAGCATCAGCAA and AAGTAGTGCAGCCCGGAGTA; calcitonin receptor (CTR), TGCAGACAACTCTTGGTTGG and TCGGTTTCTTCTCCTCTGGA; TNF-α, CATCTTCTCAAAATTCGAGTGACA and TGGGAGTAGACAAGGTACAACCC; IL-1β, TGCCACCTTTTGACAGTGATG and TGATGTGCTGCTGCGAGATT; IL-6, TCCAGTTGCCTTCTTGGGAC and AGTCTCCTCTCCGGACTTGT.

### Western Blotting Analysis

In order to investigate the effects of magnoflorine on early RANKL‐mediated signaling pathway, total cellular proteins (TCPs) and nuclear proteins were extracted from BMMs pre-treated for 4 h with serum-free α-MEM with or without magnoflorine followed by RANKL (50 ng/ml) stimulation for 0, 5, 10, 20, 30, and 60 min. To assess c-Fos and NFATc1 expression, total cellular proteins were extracted from BMMs cultured with M-CSF (30 ng/ml) and RANKL (50 ng/ml) in the absence or presence of 200 μM magnoflorine for 0, 1, 3, and 5 days. The total proteins and nuclear proteins of these specific time points were harvested respectively with radioimmunoprecipitation assay (RIPA) lysis buffer (Beyotime, Shanghai, China) supplemented with PMSF, protease and phosphatase inhibitor (Sigma-Aldrich) and with a nuclear protein extraction commercial kit (Beyotime, Shanghai, China). The lysate was centrifuged for 15 min at 12,000 × g, and the concentration of proteins in the supernatant was determined with a bicinchoninic acid assay (Biosharp Life Sciences, China) per manufacturer’s instructions. Equal amounts of the protein lysates (20 mg) were separated by electrophoresis on 10% SDS-PAGE and then transferred to PVDF membranes (Millipore, Bedford, MA). After blocking in 5% (w/v) skim milk in TBST (0.1% Tween 20 in TBS) at room temperature for 1 h, the membranes were incubated overnight at 4°C with primary antibodies diluted 1:1,000 including p38, p-p38, JNK, p-JNK, ERK, p-ERK, NF-κB-p65, p-NF-κB-p65, IκBα, p-IκBα, TAK1, p-TAK1, c-fos, NFATc1, β-actin, and histone H3. After incubating at room temperature for 1 h with appropriate secondary antibodies, antibody reactivity and protein bands were detected and quantified by exposure in an Odyssey V3.0 image scanner (LI-COR, Inc., Lincoln, NE).

### Statistical Analysis

All data were from at least three independent experiments and are presented as means ± standard deviation (SD). Statistical analyses were performed by combining one-way analysis of variance (ANOVA) and Student’s *t* tests using SPSS 22.0 software (SPSS Inc.). Differences were considered to be statistically significant at *P < 0.05, **P < 0.01 and ***P < 0.001.

## Results

### Administration of Magnoflorine Inhibited Ti Particle-Mediated Osteolysis and Inflammatory Factor Release *In Vivo*

**Figure 1:**
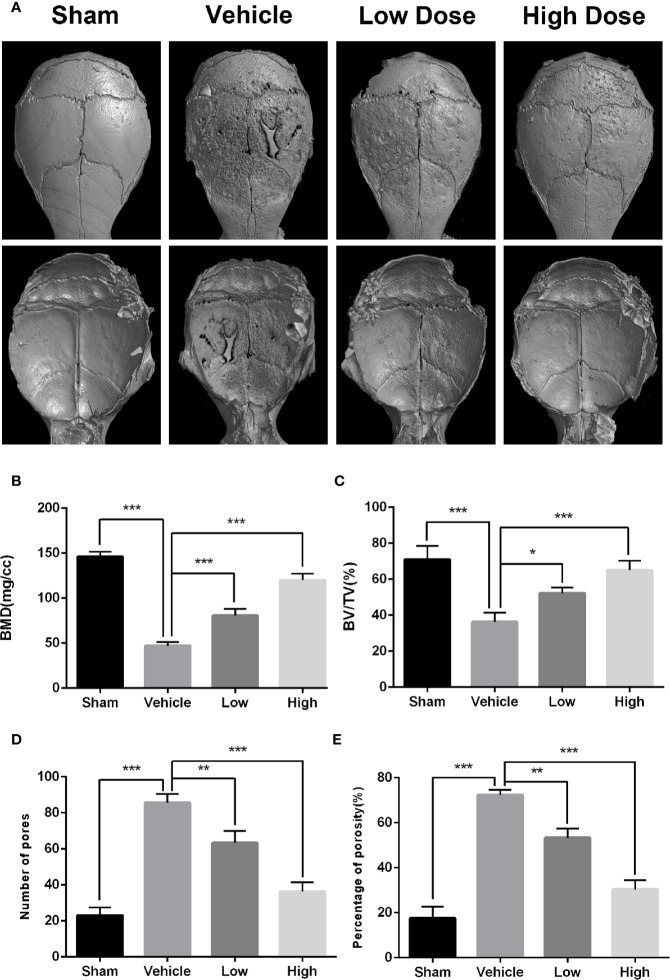
Magnoflorine ameliorates osteolysis induced by Ti particle in murine calvarias. **(A)** Representative images of three dimensional micro-CT reconstructions. Morphometric analyses of bone mineral density **(B)**, bone volume/total volume ratio **(C)**, numbers of pores **(D)**, and the percentage of porosity **(E)** within the regions of interest. Data are expressed as the mean ± SD (*P < 0.05, **P < 0.01, ***P < 0.001).

**Figure 2:**
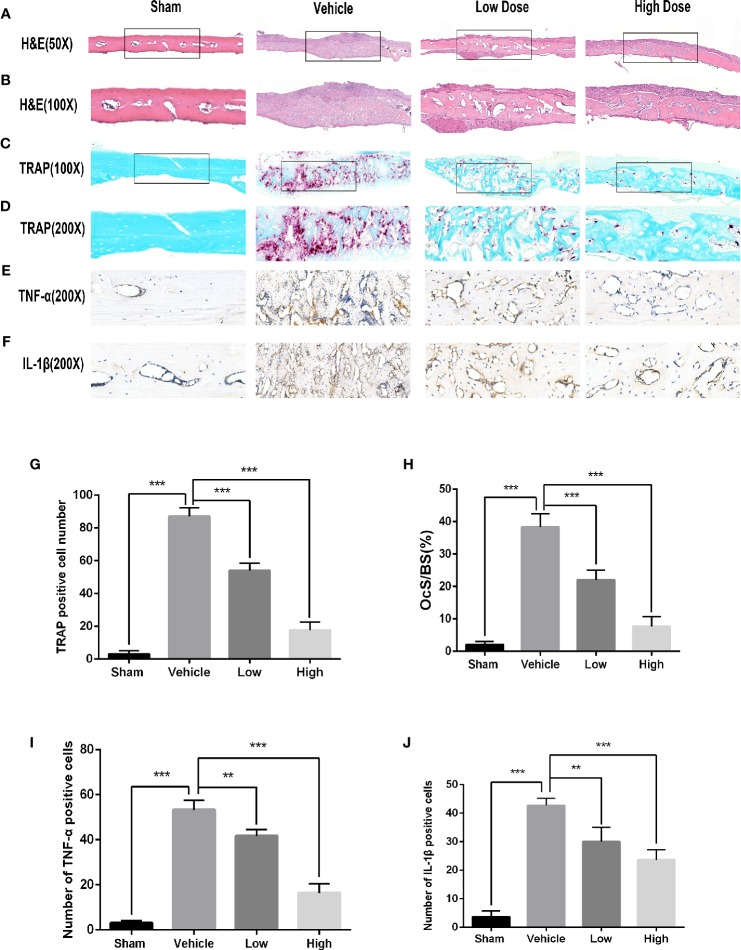
Histological evaluation of the *in vivo* inhibition of titanium particle-induced bone loss and proinflammatory cytokine expression by magnoflorine. **(A–D)** Representative images of H&E (x50 and x100) and TRAP (x100 and x200) staining indicated the decreased osteolytic region and TRAP-positive OCs in the magnoflorine-treated groups. **(E, F)** Representative images of IHC staining for TNF-α and IL-1β. Histomorphometric analyses of the numbers of TRAP‐positive osteoclasts **(G)**, OcS/BS **(H)**, and numbers of TNF-α-positive **(I)** and IL-1β-positive **(J)** cells in the calvarial bone sections were conducted. The data are presented as the mean ± SD (**P < 0.01, ***P < 0.001).

To determine if magnoflorine is protective against pathological osteolysis, we first assessed calvarial bone loss in mice treated with Ti particles. Three-dimensional reconstructions of micro-CT images revealed considerable bone erosion, with large deep pits on the calvarial surface, occurred in the Ti group (**Figure 1A**). Treatment with magnoflorine on the other hand, dose-dependently alleviated the degree of bone resorption with less bone erosion in the high-dosage group than in the low-dosage group (**Figure 1A**), as demonstrated by quantitative analyses of bone morphometric parameters (**Figures 1B–E**). Notably, craniums treated with Ti particles exhibited marked decreases in BMD and BV/TV, with increases in the number of pores as well as percentage of porosity compared with those from the sham controls. These changes were mitigated by administration of magnoflorine. Results from H&E and TRAP staining similarly revealed profound calvarial bone loss induced by Ti particles as well as diffuse infiltration of inflammatory cells, such as macrophages and lymphocytes, and abundant TRAP-positive osteoclasts along the surface of the eroded bone (**Figures 2A–D**). The Ti particle-treated group had a higher number of osteoclasts and greater OcS/BS (**Figures 2G, H**). Consistent with the micro-CT findings, magnoflorine administration dose-dependently reduced in the area of bone erosion and the accumulation of osteoclasts (**Figures 2A–D, G, H**). Immunohistochemical staining revealed that the vehicle-treated group had more TNF-α- and IL-1β-positive cells in cranial tissue than the sham group (**Figures 2E, F**). Nonetheless, the number of TNF-α- and IL-1β- positive cells was considerably suppressed by magnoflorine treatment, indicating the alleviated release of inflammatory factors (**Figures 2I, J**). Together, the *in vivo* results demonstrated that magnoflorine is protective against Ti particle-mediated osteolysis by suppressing osteoclast activity and inflammation.

### Magnoflorine Dose-Dependently Suppressed Osteoclast Differentiation Without Producing Cytotoxicity

**Figure 3:**
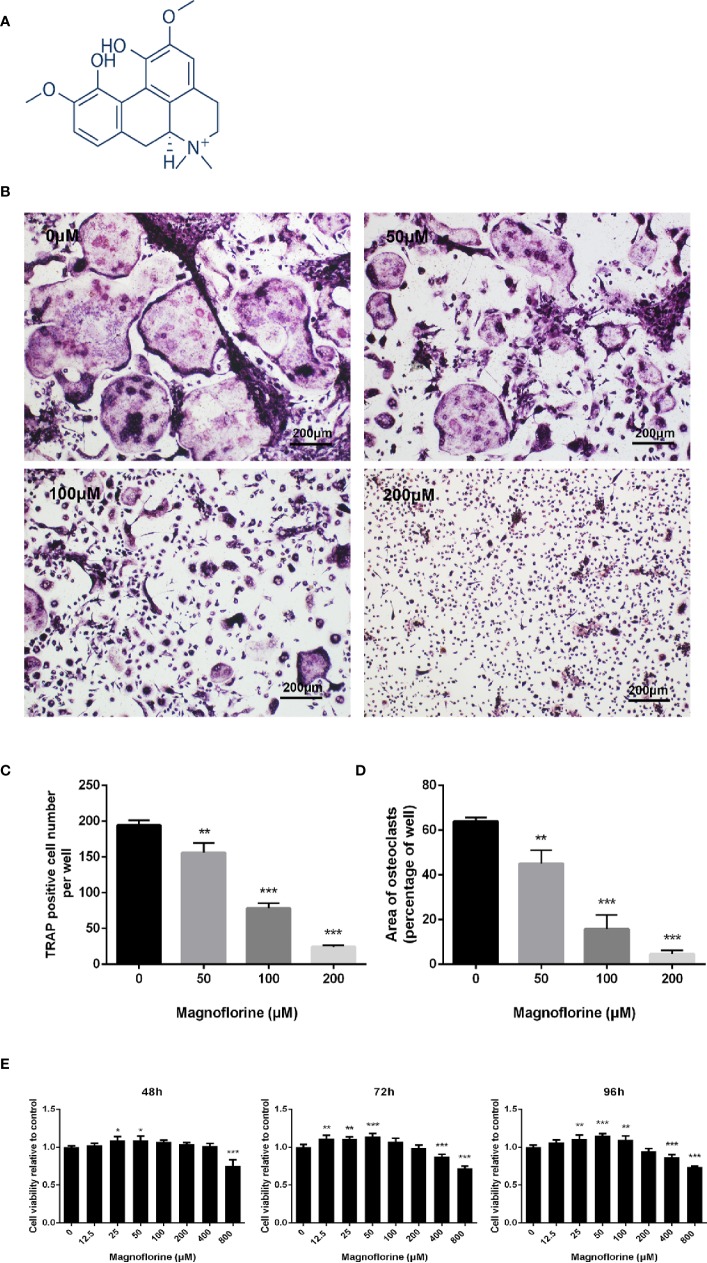
Magnoflorine dose-dependently inhibits RANKL-mediated osteoclastogenesis without cytotoxicity. **(A)** The chemical structure of magnoflorine (C_20_H_24_NO_4_, ≥98% purity, MW 342.41). **(B)** BMMs were treated with M-CSF (30 ng/mL), RANKL (50 ng/ml), and the indicated concentrations of magnoflorine for 5 days before they were fixed with 4% paraformaldehyde and stained for TRAP. The numbers **(C)** and areas **(D)** of TRAP‐positive osteoclasts were counted. **(E)** The viability of BMMs incubated with 30 ng/ml M-CSF and magnoflorine for various times was evaluated with CCK‐8 assay. Magnoflorine treatment at concentration of 200 μM did not exhibited cellular toxicity. (*P < 0.05, **P < 0.01, ***P < 0.001).

The chemical structure of magnoflorine has been shown in **Figure 3A**. The eﬀect of magnoflorine on the RANKL‐stimulated differentiation of BMMs into osteoclasts was then investigated *in vitro*. As shown in **Figure 3B**, in the control group, BMMs differentiate into numerous TRAP-positive osteoclasts that are multinucleated, well spread, and “pancake”-shaped. However, the number and area of osteoclasts exposed to magnoflorine with different dose (50, 100, and 200 μM) were dose-dependently decreased (**Figures 3B–D**). The formation of mature osteoclasts was reduced approximately 20% with 50 μM magnoflorine and was almost completely inhibited with 200 µM magnoflorine (**Figures 3B–D**). Furthermore, to exclude the possibility that the reduced osteoclast differentiation was due to cytotoxicity, we assessed the viability of the BMMs with the CCK-8 assay. As demonstrated in **Figure 3E**, no cytotoxicity effect was detected on BMM precursors after treatment with magnoflorine at concentrations of up to 200 μM, the concentration at which magnoflorine effectively suppressed osteoclastogenesis. Collectively, these results suggest that magnoflorine inhibits osteoclastogenesis without inducing cytotoxicity.

### Magnoflorine Inhibited the Formation of the Podosomal Actin Belt and the Fusion of BMM Precursors

**Figure 4:**
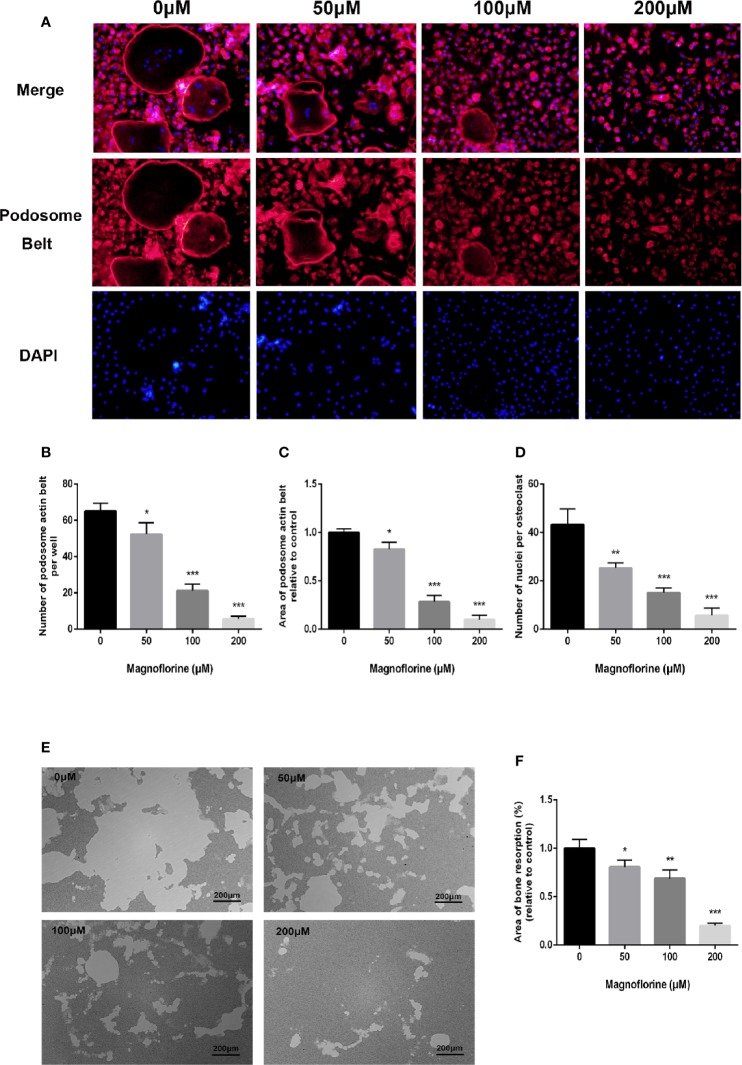
Magnoflorine impairs the formation of podosome actin belts as well as the fusion of osteoclast precursors *in vitro*, and inhibits OCs-mediated bone resorption activity. **(A)** Representative images of staining for podosomal actin belts in cultured osteoclasts in the presence of magnoflorine. BMMs were incubated with magnoflorine, RANKL (50 ng/ml), and M-CSF (30 ng/ml) for 5 days and then the mature osteoclasts were fixed and stained with rhodamine‐conjugated phalloidin and DAPI. **(B–D)** Numbers and areas of podosomal actin belts and numbers of nuclei per osteoclast. **(E)** Representative images showing resorption of BMMs cultured with or without magnoflorine on hydroxyapatite-coated Osteo assay plates. **(F)** The area of bone resorption in each group were quantified. The data are presented as the mean ± SD (*P < 0.05, **P < 0.01, ***P < 0.001).

A well‐formed cytoskeletal podosomal actin belt in the periphery of osteoclasts is an indicator of precursor cell fusion (Touaitahuata et al., 2016). Therefore, we examined whether this was influenced by magnoflorine. The immunofluorescence results showed osteoclasts exhibited a discernible and well-shaped podosomal actin belt in the RANKL‐only treated group (**Figure 4A**), whereas those treated with magnoflorine exhibited disrupted podosomal actin belts, with dose-dependent decreases in both size and number (**Figures 4B, C**). Notably, cells treated with higher concentrations of magnoflorine were primarily mononucleated, further indicating defects in precursor cell fusion (**Figure 4D**). Collectively, these findings indicated that magnoflorine treatment remarkably impaired podosomal actin belt formation in osteoclasts and inhibited osteoclast precursor cell fusion *in vitro*.

### Magnoflorine Attenuated Osteoclastic Bone Resorption Activity

Considering that the formation of an intact podosomal actin belt is indispensable to the subsequent formation of well-polarized F‐actin ring which is also critical for osteoclastic bone resorption activity (Takito et al., 2018), we therefore investigated whether magnoflorine could inhibit osteoclastic bone resorption. RANKL‐only treated groups showed extensive resorption on the surfaces of hydroxyapatite-coated culture dishes. By contrast, the sizes of the resorption areas were markedly and dose-dependently decreased by magnoflorine (**Figures 4E, F**). The area of bone resorption was reduced to 80% and 65% by 50 and 100 μM magnoflorine, respectively, and groups treated with 200 μM magnoflorine exhibited only small resorption pits that were randomly scattered around the dish (**Figures 4E, F**).

### Magnoflorine Suppressed Osteoclast-Specific Gene Expression

**Figure 5:**
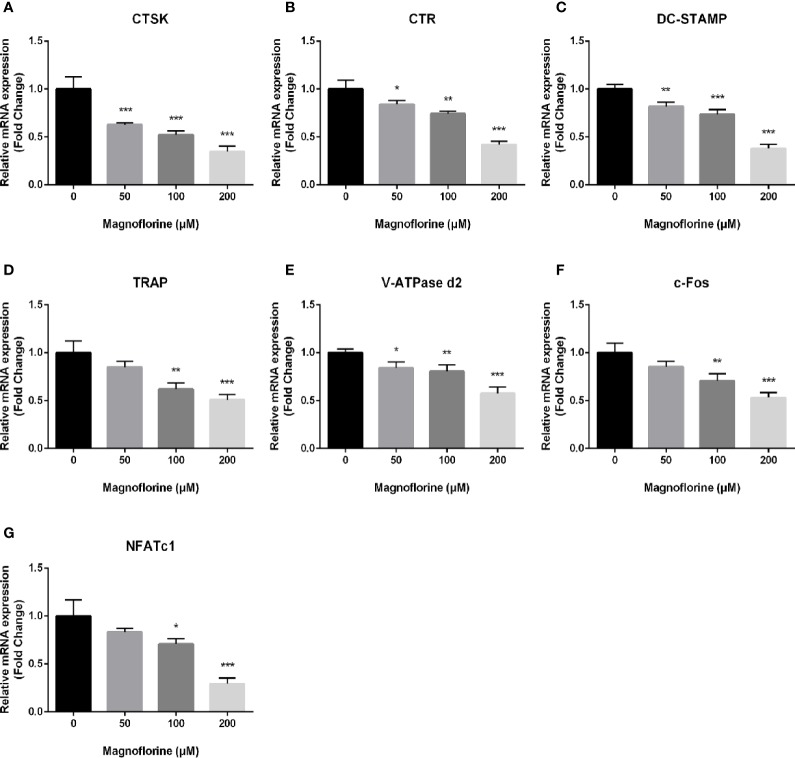
Magnoflorine downregulates expression levels of osteoclast-specific genes. qRT-PCR was conducted using RNA extracted from BMM-derived osteoclasts stimulated for 5 days with 30 ng/ml M-CSF and 50 ng/ml RANKL without or with magnoflorine. **(A–G)** The expression of osteoclast marker genes including CTSK, TRAP, CTR, NFATc1, c-fos, DC-STAMP and V-ATPase d2 were normalized to the housekeeping gene GAPDH. All experiments were performed at least three times (*P < 0.05, **P < 0.01, ***P < 0.001).

RANKL-mediated osteoclast differentiation and function are associated with the upregulation on expression level of specific marker genes. To unveil the anti‐osteoclastic effect of magnoflorine, we analyzed and quantified the expression of osteoclast-related genes in the mRNA level by qRT-PCR. The expression of genes encoding the key transcription factors NFATc1 and c-Fos, which are responsible for initiating osteoclast differentiation (Gohda et al., 2005; Monje et al., 2005), was dose‐dependently decreased in cells treated with magnoflorine in comparison with that in cells treated only with RANKL (**Figures 5A–G**). Furthermore, other osteoclast-specific markers such as CTR, CTSK, TRAP, V-ATPase d2, and DC‐STAMP demonstrated similar expression trend as NFATc1 and c-fos, on account that the genes encoding these are transcriptionally co-regulated by NFATc1 and c-fos. These data provided further evidence that magnoflorine suppresses osteoclast differentiation and bone resorption.

### Magnoflorine Alleviated Inflammation Induced by Ti Particles *In Vitro*

**Figure 6:**
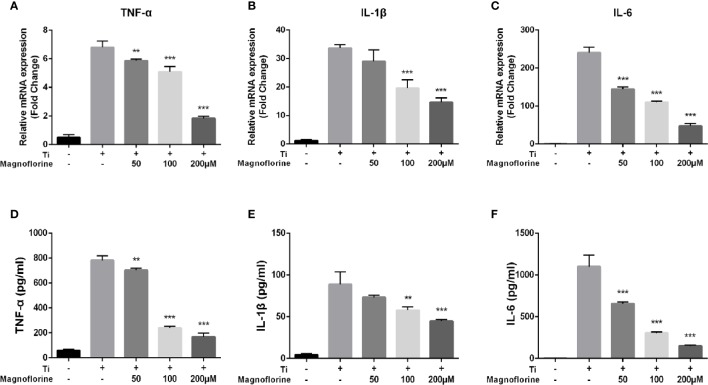
Magnoflorine alleviates Ti particle-mediated inflammation *in vitro*. BMMs were incubated for 24 h with Ti particles in the absence or presence of magnoflorine. Quantitative real-time PCR for gene expression **(A–C)** and ELISAs of culture supernatants **(D–F)** to assess levels of proinflammatory cytokines. Data are presented as mean ± SD (**P < 0.01, ***P < 0.001 versus Ti particles alone).

We performed qRT-PCR and ELISA to examine whether magnoflorine influences the release of proinflammatory cytokines induced by Ti particles. As shown in **Figures 6A–F**, TNF-α, IL-1β, and IL-6 were increased at both the mRNA and protein levels in BMMs exposed to Ti particles. Notably, treatment with magnoflorine dose-dependently attenuated this proinflammatory effect. These findings were consistent with the *in vivo* results, showing that magnoflorine administration effectively alleviates inflammatory response in Ti particle-stimulated BMMs.

### Magnoflorine Depressed the Osteoclastogenesis by Inhibiting TAK1 Phosphorylation and Downstream Activation of MAPK and NF-κB Signalings

**Figure 7:**
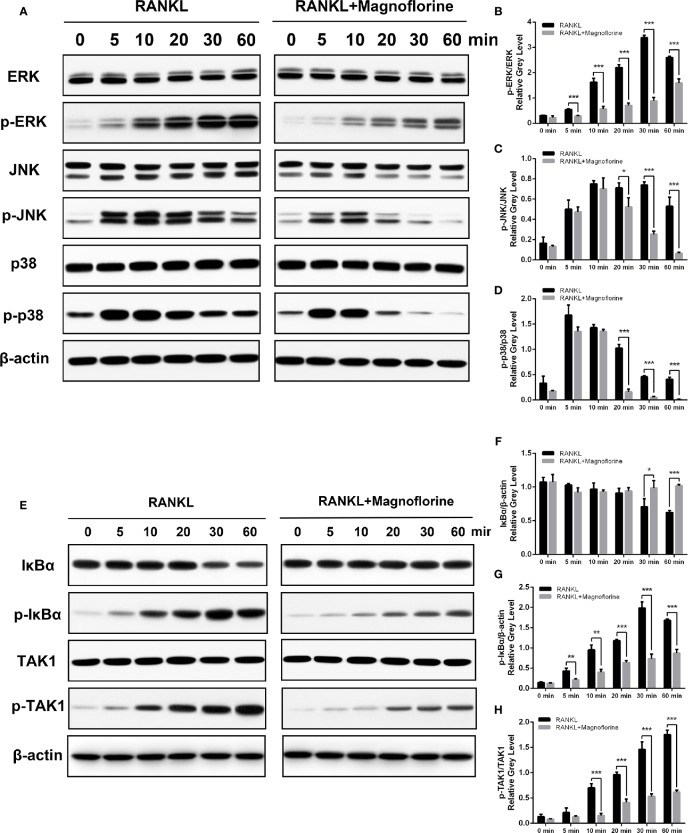
Magnoflorine inhibits osteoclast formation and function by specifically impairing TAK1 phosphorylation and downstream activation of MAPK and NF-κB signalings. **(A, E)** BMMs were pretreated for 4 h without or with 200 µM magnoflorine and then treated with 50 ng/ml RANKL for the indicated time points. Cell lysates were analyzed by Western blot to assess protein and phosphorylation levels. **(B–D)** The levels of phosphorylated MAPK signaling proteins were quantified and normalized to total levels of the corresponding proteins. **(F–H)** The levels of phosphorylated and total IκBα were normalized to that of β-actin, whereas levels of phosphorylated TAK1 were normalized to total corresponding protein levels. Data are presented as mean ± SD (*P < 0.05, **P < 0.01, ***P < 0.001).

**Figure 8:**
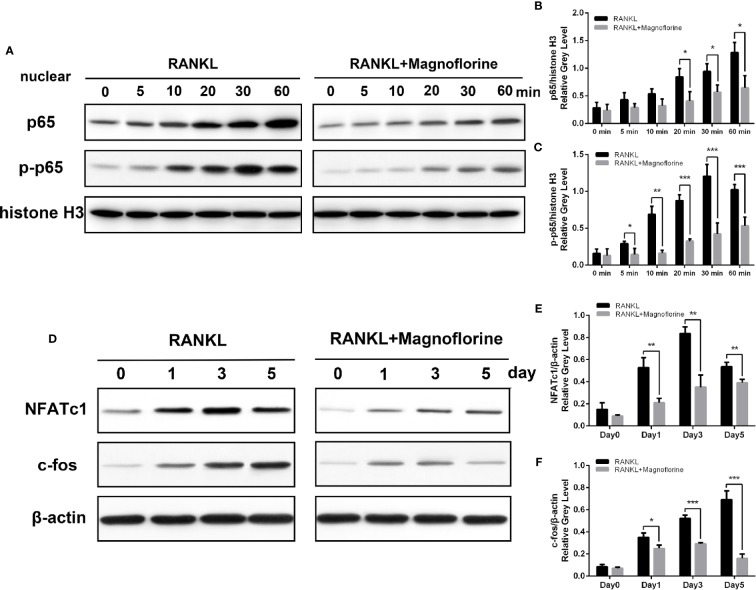
Magnoflorine suppresses osteoclast differentiation *via* the NF-κB pathway. **(A)** BMMs were pretreated for 4 h without or with 200 µM magnoflorine and then treated with 50 ng/ml RANKL for the indicated time points. Nuclear protein was isolated and analyzed by Western blot with specific antibodies against NF-κB p65 and p-NF-κB p65. **(B, C)** The levels of phosphorylated and total NF-κB p65 were quantified and normalized to histone H3. **(D–F)** BMMs were stimulated for 0, 1, 3, and 5 days with M-CSF (30 ng/ml) and RANKL (50 ng/ml) with or without 200 µM magnoflorine, and protein levels of c-Fos and NFATc1 in cell lysates, normalized to that of β-actin, were measured. Data are presented as mean ± SD (*P < 0.05, **P < 0.01, ***P < 0.001).

To further clarify the underlying molecular mechanism through which magnoflorine inhibits the formation and function of osteoclasts, we performed Western blotting to assess RANKL-induced signaling pathways, in which MAPK and NF-κB signalings are known to play pivotal roles in RANKL-mediated osteoclastogenesis (Ghosh and Karin, 2002; Ikeda et al., 2004). BMMs were stimulated with 50 ng/ml RANKL for various times, and the protein and phosphorylation levels of various members of these signaling pathways were measured. The phosphorylation of p38, ERK1/2, and JNK peaked within 5, 30, and 10 min, respectively, after RANKL stimulation (**Figures 7A–D**). However, a 4-h pretreatment with magnoflorine suppressed the activation of these signaling proteins. Moreover, RANKL stimulated the phosphorylation and translocation of NF-κB p65, and this activation was also suppressed by magnoflorine treatment (**Figures 8A–C**). NF-κB p65 activation and translocation are regulated by IκBα phosphorylation and degradation. RANKL stimulation resulted in transient phosphorylation of IκBα in control cells, which peaked at 30 min and decreased by 60 min after treatment. This effect was mitigated by pretreatment with magnoflorine (**Figures 7E, G**). The transient phosphorylation was accompanied by IκBα degradation 30 min after RANKL administration, which was also suppressed by magnoflorine, resulting in the activation of the NF-κB pathway (**Figures 7E, F**). The activation of both MAPK and NF-κB signalings were primarily due to TAK1 activation, as the phosphorylation of TAK1 was also suppressed in the presence of magnoflorine (**Figures 7E, H**). **Figures 8D–F** showed that the expression of NFATc1 and c-Fos increased gradually, consistent with activation of MAPK and NF-κB signalings, but were suppressed by administration of magnoflorine in a time-dependent manner.

## Discussion

Total hip arthroplasty is extraordinarily effective for many end-stage hip joint diseases and is commonly used treatment worldwide (Bohler et al., 2019). However, the incidences of aseptic loosening secondary to wear particle-induced periprosthetic osteolysis (PPO), the primary cause of arthroplasty failure, continue to escalate (Jiang et al., 2013; Cordova et al., 2014; Goldman et al., 2019). PPO is a serious and inevitable problem that shortens the lifespan of prosthesis and worsens the life quality of patients, ultimately resulting in the need for an expensive and complicated revision surgery (Abu-Amer et al., 2007; Gwam et al., 2017). The persistent detachment of wear particles from the articulating interface stimulates a host immune response dominated by macrophages and increased release of proinflammatory cytokines, resulting in enhanced osteoclastogenesis for bone resorption at the expense of the formation of bone-forming osteoblasts (Gallo et al., 2013; Nich et al., 2013; Athanasou, 2016; Hallab, 2016). In addition, despite advances in implant design, biomaterial properties and surgical techniques, the wear particles generated from implant surfaces is inevitable (Mutimer et al., 2010; Bryan et al., 2019). Therefore, osteoclasts and inflammation represent therapeutic targets to prevent wear debris-mediated bone loss after total hip arthroplasty. Among the myriad therapeutic effects of magnoflorine (Zheng and Wang, 2001; Min et al., 2006; El Tahir, 2008; Patel and Mishra, 2012; Guo et al., 2018), it is the anti-inflammatory properties of this quaternary alkaloid that may provide protection against osteolysis in patients with prostheses, as inflammation impacts the formation and function of osteoclasts.

Here, we showed that magnoflorine was protective against Ti particle-induced calvarial osteolysis *in vivo*. This protection was due to the inhibition of RANKL-induced MAPK (p38, ERK1/2, and JNK) and NF-κB signalings during osteoclast formation and bone resorption, as well as attenuation of the release of Ti particle-mediated proinflammatory cytokines. This protective effect was observed both by micro-CT and by histologic and immunohistochemical analyses. Our results provide evidence that magnoflorine dose-dependently suppressed various measures of bone loss and resorption. In addition to a significant reduction in the erosion area, magnoflorine reduced the number of osteoclasts and infiltration of inflammatory cells.

The results from *in vitro* experiments further clarified the anti-osteoclastogenic and anti-inflammatory properties of magnoflorine. Magnoflorine suppressed the differentiation of osteoclasts from BMM precursors and impeded bone resorption. These effects were observed in the absence of any cytotoxicity. We also found that precursor cell fusion was impaired, which was attributed to a defect in the formation of podosome actin belts. The effect of magnoflorine on the development of osteoclasts was attributed to reduced expression of a variety of osteoclast-specific genes, including those regulating transcription of downstream marker genes (NFATc1 and c-Fos) (Yamashita et al., 2007), encoding enzymes for osteoclastic bone absorption (TRAP and CTSK) (Saneshige et al., 1995), maintaining calcium homeostasis (CTR) (Granholm et al., 2008), and precursor cell fusion (V-ATPase d2 and Dc-stamp) (Wu et al., 2009).

Having illuminated that magnoflorine exerts inhibitory effects on Ti particle-mediated osteolysis *in vivo* and RANKL-induced osteoclastogenesis *in vitro*, we further probed into the underlying molecular mechanisms. When RANKL binds to its receptor RANK on membranes of osteoclast precursors, TNF receptor-associated factor 6 (TRAF6) is recruited and activated, which combines with RANK and TAK1-binding protein 2 to phosphorylate TAK1 (Gohda et al., 2005; Sumiya et al., 2015), which in turn phosphorylates both MAPK and IκB kinases. The subsequent signaling through MAPK and NF-κB pathways is crucial for osteoclast formation (Idris et al., 2010; Park et al., 2017; Lee et al., 2018). Previous studies reported that RANKL-induced osteoclast differentiation was markedly inhibited by blockade of JNK signaling (Ikeda et al., 2004). In addition, p38 signaling pathway regulates the transcription of downstream target genes associated with osteoclast formation (Matsumoto et al., 2000). On the other hand, activation of ERK regulates osteoclast survival and apoptosis and maintains cell polarity during bone resorption (Nakamura et al., 2003). We found that magnoflorine suppressed the RANKL-mediated phosphorylation level of these MAPK signaling pathway components. Similarly, magnoflorine inhibited the activation of NF-κB, which involves nuclear translocation and phosphorylation of p65 and the phosphorylation and degradation of IκBα (Jimi and Ghosh, 2005; Keating et al., 2007; Xu et al., 2009). We also found that p-TAK1 was attenuated after the intervention of magnoflorine, which might account for the inactivation of both MAPK and NF-κB signalings. Accordingly, the expression of NFATc1, a downstream target of NF-κB (Takayanagi et al., 2002), was reduced by magnoflorine. Immediately after NF-κB translocates to the nucleus, it binds to the promoter of NFATc1 to induce auto-amplification (Asagiri et al., 2005; Yamashita et al., 2007). This process requires the transcription factor c-Fos, which is regulated by MAPK signaling pathways (Lucas et al., 1998; Takayanagi et al., 2002; Huang et al., 2006). As we hypothesized, inhibition of NF-κB and MAPK activation by magnoflorine downregulated the protein levels of NFATc1 and c-Fos, and subsequently inhibited the expression of NFATc1-regulated osteoclastic marker genes including CTSK, TRAP, CTR, V-ATPase d2, and Dc-stamp, resulting in impaired osteoclast formation.

The failure of periprosthesis bone integration is partially attributed to chronic inflammation, which is stimulated by particles shed from the prosthesis. The recruitment and activation of macrophages, fibroblasts, and lymphocytes results in increases in various inflammatory mediators that exacerbate osteoclastic bone erosion (Goodman, 2007; Suzuki et al., 2007; Bechtel et al., 2016). In this work, we can observe through H&E staining extensive infiltration of macrophages and lymphocytes in Ti-treated group whereas not in the magnoflorine-treated group, indicating an excellent anti-inflammatory effect exhibited by magnoflorine. As expected, the immunohistochemistry staining showed TNF-α and IL-1β positive cells significantly decreased after the administration of magnoflorine, which was in parallel with dose-dependently alleviated mRNA and protein levels of inflammatory factors *in vitro*, indicating that magnoflorine can protect against PPO by inhibiting localized inflammation.

There are several limitations of this study that should be noted. First, ultra-high-molecular-weight polyethylene wear particles more commonly are the cause of implant failure than Ti particles (Tipper et al., 2006; Qu et al., 2013). However, it has been documented that both particle types comparably activate osteoclasts and trigger pronounced osteolytic effects (von Knoch et al., 2004; Taki et al., 2005). Thus, it is reasonable to establish the osteolytic animal models involving both titanium and ultra-high-molecular-weight polyethylene wear particles. Second, osteolysis was examined in murine calvarias, which differ from long bone structures in terms of bone microarchitecture and mechanical load. Furthermore, as noted by von Knoch et al., particles are liberated continuously from the prosthesis surface in patients rather than as a single bolus, as in the experimental model (von Knoch et al., 2004). Therefore, an animal model with continuous exposure lower concentrations of Ti particles may be more desirable in future studies. What’s more, in this study, we adopted the method of local injection, which may not apply to humans, instead of oral administration. Therefore, surface modification of implants may be a preferable way, which avoids the limitation of local injection and can reduce side effects to some extent and improve the efficacy by the local release of natural compound. In future research, we will establish a model, treating local osteolysis by implanting titanium rods modified with magnoflorine on the surface into the medullary cavity, as a basis for future human studies. Finally, PPO *in vivo* is a complicated course involving functional balance between osteoblastic bone formation and osteoclastic bone resorption (Purdue et al., 2007), and bone regeneration also plays a crucial part in protecting against osteolysis (Saleh et al., 2004). Whether magnoflorine can promote osteogenic differentiation and thus enhance bone regeneration should be explored in further studies.

In conclusion, the results presented here demonstrate that magnoflorine possesses unexpected properties that attenuate Ti particle-induced inflammatory osteolysis *in vivo* and suppress inflammatory responses and RANKL-induced osteoclastogenesis *in vitro*. Furthermore, the inhibitory effects were accomplished through the inhibition of TAK1 phosphorylation and downstream activation of MAPK and NF-κB signaling pathways. Altogether, these findings indicate that magnoflorine may potentially be used to prevent or treat periprosthetic osteolysis as well as other osteolytic bone diseases associated with chronic inflammation and imbalanced osteoclast formation.

## Data Availability Statement

All datasets generated for this study are included in the article/supplementary material.

## Ethics Statement

The animal study was reviewed and approved by Animal Care Committee of Shanghai Jiao Tong University.

## Author Contributions

ZS, JZ, and WW contributed conception and design of the research. ZS, JZ, and XJ performed *In Vitro* experiments. ZS, WW, and QW performed the animal experiments. ZS, JZ, and WW organized the database and performed the statistical analysis. ZS and JZ wrote the manuscript. YM and DY revised the manuscript and supervised the whole study.

## Conflict of Interest

The authors declare that the research was conducted in the absence of any commercial or financial relationships that could be construed as a potential conflict of interest.
